# Adverse events following COVID‐19 mRNA vaccines: A systematic review of cardiovascular complication, thrombosis, and thrombocytopenia

**DOI:** 10.1002/iid3.807

**Published:** 2023-03-17

**Authors:** Farah Yasmin, Hala Najeeb, Unaiza Naeem, Abdul Moeed, Abdul Raafe Atif, Muhammad Sohaib Asghar, Nayef Nimri, Maryam Saleem, Dhrubajyoti Bandyopadhyay, Chayakrit Krittanawong, Mohammed Mahmmoud Fadelallah Eljack, Muhammad Junaid Tahir, Fahad Waqar

**Affiliations:** ^1^ Department of Internal Medicine Dow University of Health Sciences Karachi Pakistan; ^2^ Division of Nephrology and Hypertension, Mayo Clinic Rochester Minnesota USA; ^3^ Department of Cardiovascular Medicine University of Cincinnati Cincinnati Ohio USA; ^4^ Department of Cardiovascular Medicine Westchester Medical Center Valhalla New York USA; ^5^ Department of Cardiovascular Medicine Baylor College of Medicine Houston Texas USA; ^6^ Department of Community Medicine University of Bakht Alruda Ed Dueim Sudan; ^7^ Department of Radiology Pakistan Kidney and Liver Institute and Research Center Lahore Pakistan

**Keywords:** cardiovascular complications, COVID‐19 vaccines, genes, infection, Pfizer−BioNTech, public health, SARS‐CoV‐2

## Abstract

**Background and Objectives:**

Since publishing successful clinical trial results of mRNA coronavirus disease 2019 (COVID‐19) vaccines in December 2020, multiple reports have arisen about cardiovascular complications following the mRNA vaccination. This study provides an in‐depth account of various cardiovascular adverse events reported after the mRNA vaccines' first or second dose including pericarditis/myopericarditis, myocarditis, hypotension, hypertension, arrhythmia, cardiogenic shock, stroke, myocardial infarction/STEMI, intracranial hemorrhage, thrombosis (deep vein thrombosis, cerebral venous thrombosis, arterial or venous thrombotic events, portal vein thrombosis, coronary thrombosis, microvascular small bowel thrombosis), and pulmonary embolism.

**Methods:**

A systematic review of original studies reporting confirmed cardiovascular manifestations post‐mRNA COVID‐19 vaccination was performed. Following the PRISMA guidelines, electronic databases (PubMed, PMC NCBI, and Cochrane Library) were searched until January 2022. Baseline characteristics of patients and disease outcomes were extracted from relevant studies.

**Results:**

A total of 81 articles analyzed confirmed cardiovascular complications post‐COVID‐19 mRNA vaccines in 17,636 individuals and reported 284 deaths with any mRNA vaccine. Of 17,636 cardiovascular events with any mRNA vaccine, 17,192 were observed with the BNT162b2 (Pfizer−BioNTech) vaccine, 444 events with mRNA‐1273 (Moderna). Thrombosis was frequently reported with any mRNA vaccine (*n* = 13,936), followed by stroke (*n* = 758), myocarditis (*n* = 511), myocardial infarction (*n* = 377), pulmonary embolism (*n* = 301), and arrhythmia (*n* = 254). Stratifying the results by vaccine type showed that thrombosis (80.8%) was common in the BNT162b2 cohort, while stroke (39.9%) was common with mRNA‐1273 for any dose. The time between the vaccination dosage and the first symptom onset averaged 5.6 and 4.8 days with the mRNA‐1273 vaccine and BNT162b2, respectively. The mRNA‐1273 cohort reported 56 deaths compared to the 228 with BNT162b2, while the rest were discharged or transferred to the ICU.

**Conclusion:**

Available literature includes more studies with the BNT162b2 vaccine than mRNA‐1273. Future studies must report mortality and adverse cardiovascular events by vaccine types.

## Graphical abstract

We aim to summarize the events of cardiac complications following the mRNA coronavirus disease 2019 vaccine, providing an in‐depth analysis of their occurrences, and their implications. The review includes 69 case reports/case series, 4 studies with data obtained from electronic medical records (hospital surveillance data, national database, VAERS/VigiBase), and 8 observational studies including prospective/retrospective cohort.

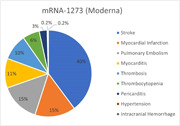

## 1 INTRODUCTION

Since the genome sequencing of the severe acute respiratory syndrome coronavirus 2 (SARS‐COV‐2), the etiologic agent of coronavirus disease 2019 (COVID‐19), in January 2020, a global race for the development of vaccines began. The swift research and development process has sparked significant concerns about the safety profile of novel coronavirus vaccines. According to a study from August 2020, 70% of the participants had safety concerns due to the rapid development of vaccines.^1^ Moreover, a scoping review conducted in September 2021 also highlighted safety concerns as the leading predictor of hesitance.^2^ These perturbations were further solidified when sporadic adverse events following immunization were reported as the large‐scale vaccination programs progressed.

A study based on the World Health Organization (WHO) database (VigiBase) documented the highest number of cardiovascular (CV) adverse events in patients receiving the BNT162b2 (Pfizer−BioNTech) COVID‐19 vaccine. This study categorized 30% and 44% of CV adverse events as severe upon vaccination by BNT162b2 (Pfizer−BioNTech) and mRNA‐1273 (Moderna), respectively. Palpitations and tachycardia were the common CV adverse events in both vaccines.^3^ Apart from this, Simone et al. reported a very low incidence of myocarditis (*n* = 15) following mRNA COVID‐19 vaccination in a large sample of participants who did not have any prior cardiac disease.^4^ Similarly, another large‐scale study conducted in the healthcare sector observed a temporal relationship of myocarditis in young males following Pfizer−BioNTech vaccination.^5^

Although the pathogenesis behind the CV manifestations remains unclear, some studies document the potential mechanism of post‐COVID‐19 vaccine CV adverse events. In genetically predisposed individuals, the mRNA vaccine might trigger an immune response resulting in the detection of mRNA as an antigen. Activation of inflammatory cascades, following the expression of cytokines by dendritic and Toll‐like receptors, results in an immunomodulatory response against the mRNA, potentially leading to myocarditis and other systemic reactions.^6^, ^7^ Similarly, postvaccination immune thrombocytopenic purpura (ITP) might be accredited to increased macrophage activity and reduced platelet production in patients with mild “compensated” thrombocytopenia or chronic, hereditary thrombocytopenia.^8^, ^9^ The distinctive feature of ITP after vaccination is the occurrence of thrombosis at uncommon sites, including the splanchnic, adrenal, cerebral, and ophthalmic veins, as concluded from the postmortem findings of patients with vaccine‐induced thrombotic thrombocytopenia (VITT).^10^

In late April 2021, officials from the Israeli Health Ministry reported the incidence of myocarditis in people receiving the BNT162b2 (Pfizer−BioNTech) vaccine.^11^ Furthermore, the Vaccine Adverse Event Report System (VAERS) database of the Food and Drug Administration (FDA) was utilized by Military.com to review the frequency of adverse events following COVID‐19 immunization; found a total of 45 cases of myocarditis, with 19 being from BNT162b2 (Pfizer−BioNTech) and 26 from mRNA‐1273 (Moderna).^12^ To discern the long‐term impact of the cardiac complications caused by the two approved COVID‐19 mRNA vaccines, the Centers for Disease Control and Prevention (CDC) has launched an investigation by surveying the patients with reported myocarditis after immunization.^13^ In this systematic review, we aim to summarize the events of cardiac complications following the mRNA COVID‐19 vaccine, providing an in‐depth analysis of their occurrences, and their implications.

## 2 MATERIALS AND METHODS

### 2.1 Search strategy

This systematic review was performed in conformity with the PRISMA guidelines.^14^ PubMed Central, Cochrane, Clinicaltrials.gov, and Scopus were searched from inception till January 2022 while preprint portals like bioRxiv, medRxiv, Authorea, and Research Square were also followed with the following search term: (COVID‐19 mRNA vaccines) AND (cardiac OR cardiovascular) AND (adverse events OR complications) AND (thrombosis OR thrombocytopenia).

Articles were first screened by title, then abstract, and finally by full text by two independent reviewers (H. N. and A. R. A.). Data extraction of relevant shortlisted articles was conducted for the following: study type, age, number of males and females, vaccine type, COVID‐19 status, comorbidities, abnormal lab parameters, CV manifestations, and clinical outcomes.

### 2.2 Inclusion and exclusion criteria

The study inclusion criteria were: (a) observational studies and case reports assessing CV complications, (b) mRNA COVID‐19 vaccine, and (c) no language restriction. The exclusion criteria were: (a) reviews, meta‐analysis, protocols, editorials, and conference abstracts, (b) non‐CV manifestations, and (c) patients with preexisting CV diseases. Relevant studies were sorted in this study based on the inclusion criteria through thematic presentation.

## 3 RESULTS

### 3.1 Literature search results

**Figure 1:**
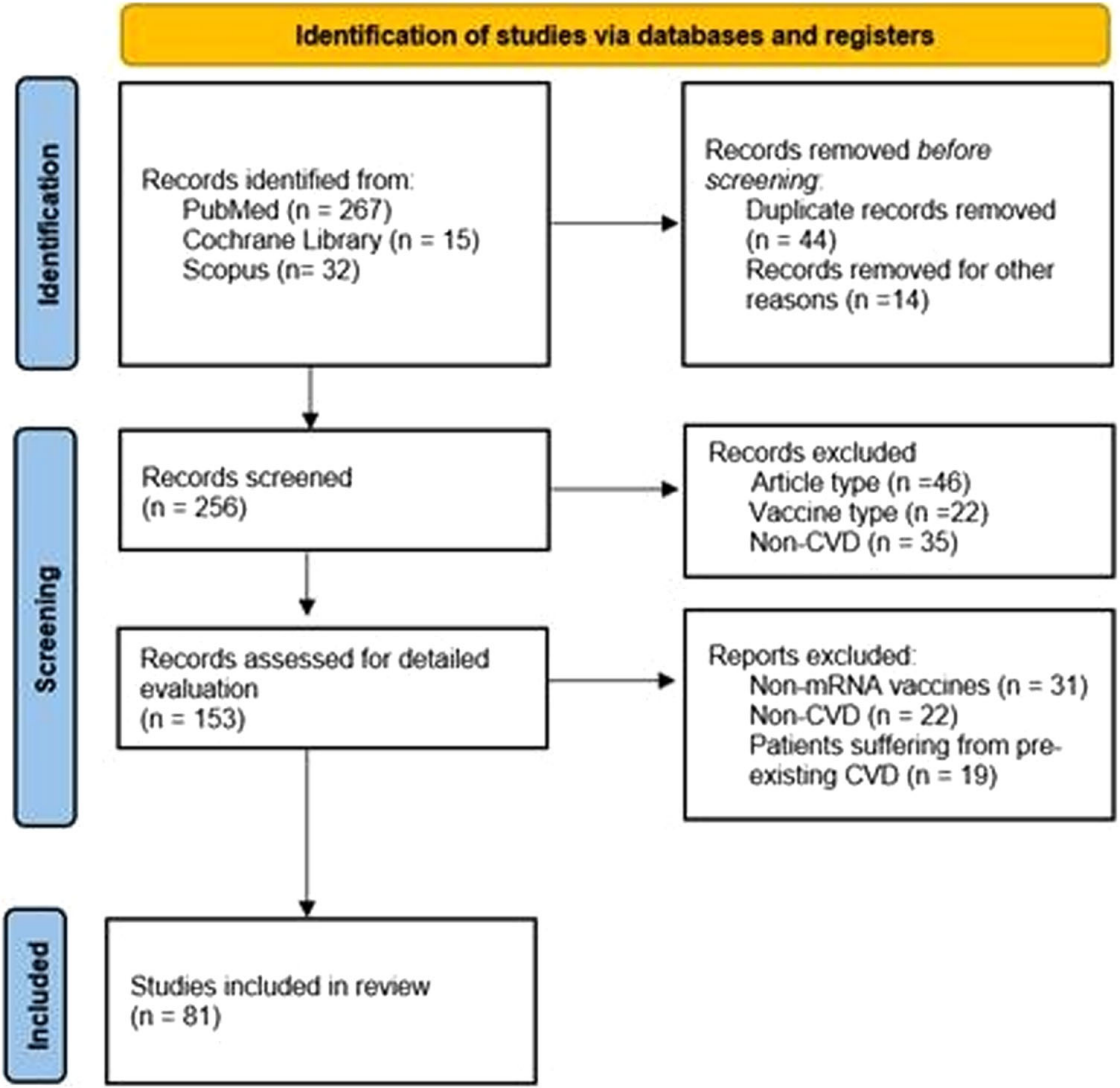
PRISMA flow chart of studies retrieved, screened, and included in this systematic review.

The search strategy and manual search yielded 314 results, of which 44 studies were removed as duplicates. Of the 256 studies screened, 103 were excluded for article types (systematic reviews, reviews, meta‐analyses, protocols, and conference abstracts), vaccine type (whole virus, protein subunit, viral vector, and DNA nucleic acid vaccines), and clinical manifestations (non‐CV presentations). A detailed evaluation of 153 studies led to the inclusion of 81 studies, as shown in Figure 1, which analyzed confirmed CV, thrombotic, and thrombocytopenic complications post‐COVID‐19 mRNA vaccination. Studies were excluded if adverse events were not specified as CV events, outcomes were not given by the type of vaccine, or presented as pregnancy‐related vascular complications.

### 3.2 Characteristics of included studies and overall results

The review includes 69 case reports/case series, 4 studies with data obtained from electronic medical records (hospital surveillance data, national database, VAERS/VigiBase), and 8 observational studies including prospective/retrospective cohorts. Of the individuals suffering from CV complications, 286 were lost to treatment.

### 3.3 CV events

A total of 17,636 CV events were reported with any mRNA vaccines. The most‐reported events in the search included pericarditis/myopericarditis, myocarditis, hypotension, hypertension, arrhythmia, cardiogenic shock, stroke, myocardial infarction (MI)/STEMI, intracranial hemorrhage, thrombosis (deep vein thrombosis [DVT], cerebral venous thrombosis [CVT], arterial or venous thrombotic events, portal vein thrombosis, coronary thrombosis, microvascular small bowel thrombosis) and pulmonary embolism. From a total of 17,636 CV complications documented, thrombosis was most frequently reported with any mRNA vaccine (*n* = 13,936), followed by stroke (*n* = 758), myocarditis (*n* = 511), MI (*n* = 377), pulmonary embolism (*n* = 301), and arrhythmia (*n* = 254). Cardiogenic shock was the least reported outcome with only 1 event, while changes in blood pressure were experienced by 39 individuals receiving any mRNA vaccine. Besides echocardiography, electrocardiogram (ECG) remained a common diagnostic tool in individuals receiving any COVID‐19 mRNA vaccine. ST‐elevation was the prevalent ECG finding with 0.6% in BNT162b2 (Pfizer−BioNTech) and 4.1% in mRNA‐1273 (Moderna) vaccine groups. Table 1 gives a detailed stratification of CV outcome by type of vaccines, the dosage of vaccines, and the total reported events in each vaccine type. Table 2 summarizes the major findings of the review for each mRNA vaccine, including abnormal lab values, treatment methods, onset to first symptom postvaccination, and mortality.

**Table 1:** Adverse events stratified by COVID‐19 vaccine type.

	BNT162b2 (Pfizer−	mRNA‐1273
	BioNTech)	(Moderna)
Cardiac events		
Pericarditis	68	13
Myocarditis	462	49
Myocardial infarction	310	67
Arrythmia	254	0
Cardiogenic shock	1	0
Thrombotic events	13,893	43
Thrombocytopenia events	1346	28
Vascular events		
Hypertension		
First dose	8	1
Second dose	10	0
Hypotension		
First dose	0	0
Second dose	10	
Stroke	581	177
Pulmonary embolism	236	65
Intracranial hemorrhage		
First dose	13	1
Second dose		0

Abbreviation: COVID‐19, coronavirus disease 2019.

**Table 2:** Overall summary of literature by vaccine types.

Characteristics	mRNA‐1273	BNT162b2
	(Moderna)	(Pfizer−BioNTech)
Total events	444	17,192
Sex		
Males	178	8042
Females	193	6102
Unidentified	73	3048
Case management		
Colchicine	6	30
NSAIDs^a^	7	39
Steroids^b^	15	38
ACE inhibitors or ARB^c^	4	8
Beta blockers^d^	6	13
Anticoagulant & Xa inhibitors^e^	5	13
Antiplatelet^f^	0	3
Platelet transfusion	4	15
IVIG	10	30
Diuretics^g^	3	4
Mean time between vaccine and symptom onset (days)	5.6	4.8
Mean length of hospitalization (days)	3.7	6.0
Mortality (*n*)^h^	56	228
Laboratory findings (*n* with highest values)		
d‐Dimer level >500 (ng/mL)	5	12
CK‐MB (ng/mL)	0	9
Troponin‐I	8	27
Troponin‐T	5	21
CRP levels >10 mg/L	13	50
ECG findings		
ST elevation	16	70
ST depression	1	7
PR depression	7	10
T‐wave abnormality	2	17
Bundle branch block	1	2
Sinus tachycardia	2	9

Abbreviation: ECG, electrocardiogram.

^a^ NSAIDs used in treatment include ibuprofen, aspirin, acetylsalicylic acid, ketorolac.

^b^ Steroids listed: corticosterone, prednisone, methylprednisolone, dexamethasone.

^c^ ARB/ACE inhibitors: lisinopril, ramipril, candesartan.

^d^ Beta blockers: bisoprolol, metoprolol, carvedilol.

^e^ Anticogulants/Xa inhibitors: rivaroxaban, apixaban, warfarin, enoxaparin, heparin, clexane dabigatran, lovenox.

^f^ Antiplateletes: clopidogrel, eptifibatide, ticagrelor.

^g^ Diuretics: acetazolamide, furosemide, mineralocorticoid antagonist.

^h^ *n* = 2 vaccine types unspecified.

### 3.4 Stratification by vaccine types

#### 3.4.1 mRNA‐1273 (Moderna)

**Figure 2:**
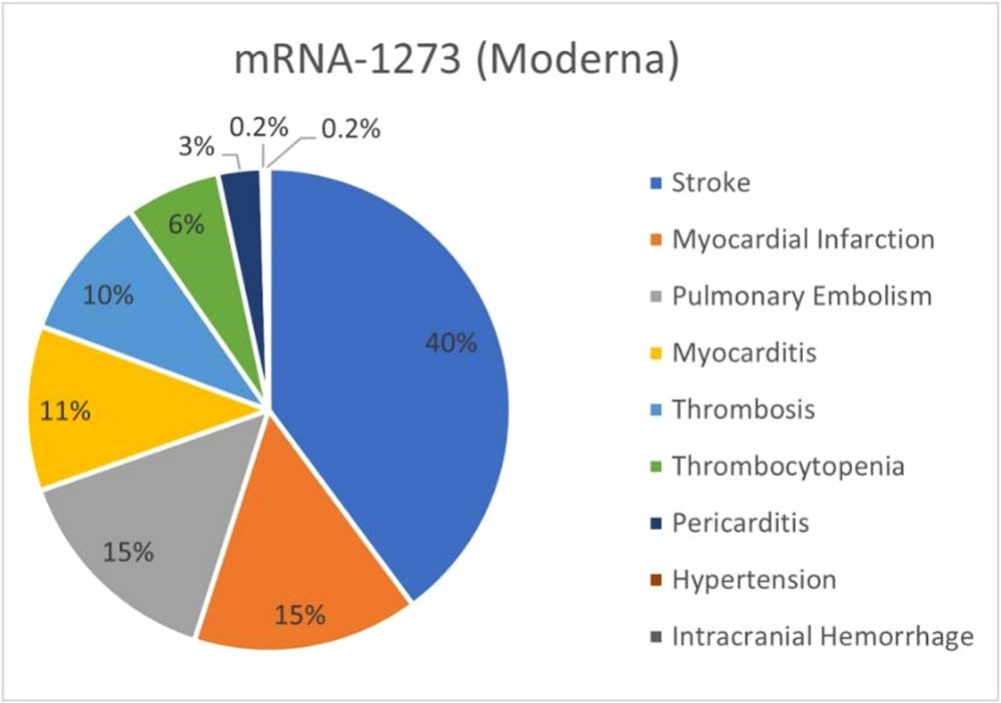
Cardiac and vascular events observed with mRNA‐1273 (Moderna) vaccine with any dose.

A smaller number of studies (*n* = 18) in the review reported 444 CV events in individuals who received the mRNA‐1273 (Moderna) vaccine at first, second, or both doses; 4 studies included both types of mRNA vaccines were previously published. Figure 2 presents the CV complications in mRNA‐1273 (Moderna) cohort. The most common complication, stroke, was reported in 39.9% of cases, followed by 15% of MI and pulmonary embolism each, and myocarditis in 11% of the cases with any dose. The remainder 19.1% of the events comprised 43 cases of thrombosis, and 13 cases of pericarditis after any dose. One event of intracranial hemorrhage and hypertension each was experienced after the first dose.

The average time between the vaccination dosage and the onset of the first symptom was 5.6 days. Individuals requiring hospitalization had a median length of 3.7 days of hospital stay. The study synthesizes data of abnormal or elevated lab values; 13 events were reported for elevated CRP levels, 5 individuals had increased Troponin T; whereas 8 cases observed an increase in Troponin I levels.

ECG and cardiac magnetic resonance imaging were the commonly used diagnostic procedures among hospitalized patients for any adverse outcomes. Data reveals 16 cases of ST‐elevations and 7 PR‐depressions, followed by ST‐depressions, T‐wave abnormalities, and bundle branch blocks.

In studies that reported treatment options steroids (*n* = 15 cases) including corticosteroids, prednisone, methylprednisolone, and dexamethasone frequently opted for. This was followed by IVIG in 10 cases, NSAIDs in 7, and colchicine in 6 cases, respectively. Analysis of literature reported mortality in 56 cases (12.6%); with the rest being discharged or transferred to ICU.

### 3.5 BNT162b2 (Pfizer−BioNTech)

**Figure 3:**
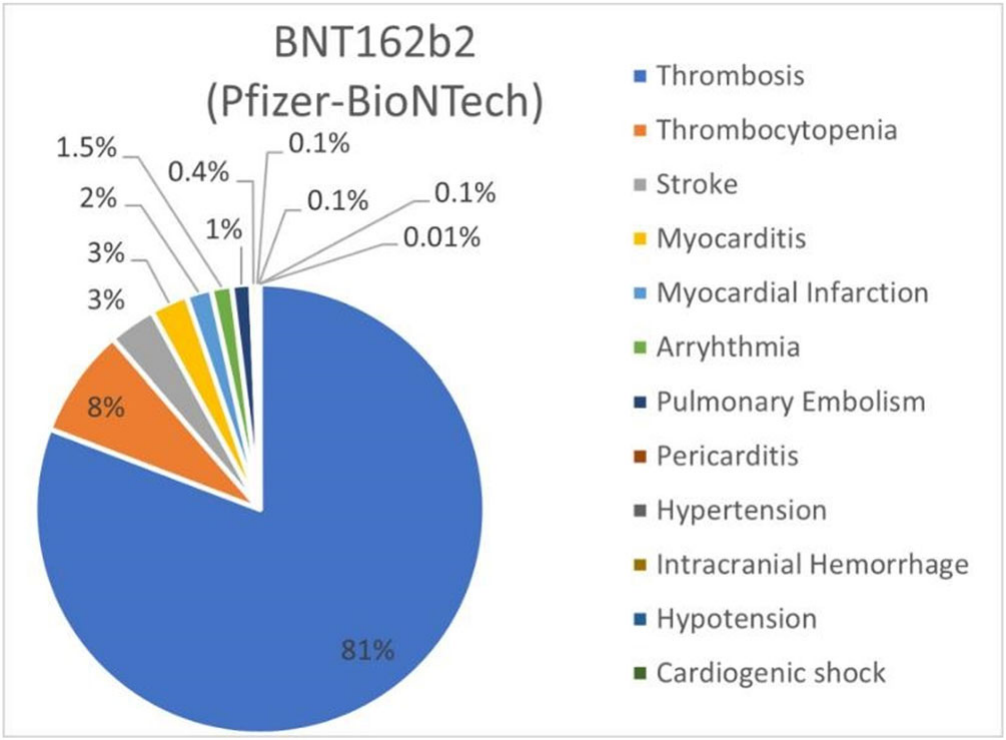
Cardiac and vascular events observed with BNT162b2 (Pfizer−BioNTech) vaccine with any dose.

A major proportion of studies (*n* = 59) in the review reported 17,192 confirmed CV events in individuals who received the BNT162b2 (Pfizer−BioNTech) vaccine, and 4 studies reporting both types of mRNA vaccines were found in published literature. With 13,893 events (80.8%), any type of thrombosis remained the most common CV outcome in this cohort. Stroke was observed in 581 cases (3.4%), and myocarditis in 462 events (2.7%), compared with 68 events of pericarditis with the first or second dose of BNT162b2 (Pfizer−BioNTech) vaccine. Observed with BNT162b2 (Pfizer−BioNTech) vaccine only, arrhythmia, MI, and pulmonary embolism accounted for 1.5%, 1.8%, and 1.4% of events, respectively. Eight events of hypertension were reported after the first BNT162b2 (Pfizer−BioNTech) vaccine dose. Hypotension and hypertension were observed after the second dosage in 10 individuals each. Cardiogenic shock (*n* = 1) was the least reported outcome in the cohort. Figure 3 summarizes the CV events in the cohort.

Excluding studies that reported median days for the onset of symptoms and vaccine dosage, the average time to the first CV symptom was 4.8 days, while the average hospital stay was recorded to be 6 days in individuals requiring hospitalization. Of the studies reporting elevated lab parameters postvaccination, increased CRP levels were observed in 50 individuals. Abnormal Troponin‐I and Troponin‐T levels were recorded in 27 and 21 cases, respectively. Elevated d‐dimer levels (>500 ng/L) were reported in 12 cases, followed by 9 cases of increased CK‐MB levels.

The most prevalent ECG finding was ST elevation in 70 individuals, followed by a T‐wave abnormality in 17 individuals. Bundle branch blocks and sinus tachycardia were the least observed findings in individuals receiving the BNT162b2 (Pfizer−BioNTech) vaccine. Critical cases were managed mostly with NSAIDS (*n* = 39) and steroids (*n* = 38). Colchicine and IVIG were reported to be used in 30 cases each. Antiplatelet therapy and diuretics were the least used treatment options. Mortality was reported in 228 cases (1.3%), with the rest of the population being discharged or transferred to critical care.

### 3.6 Treatment options and diagnostics

**Figure 4:**
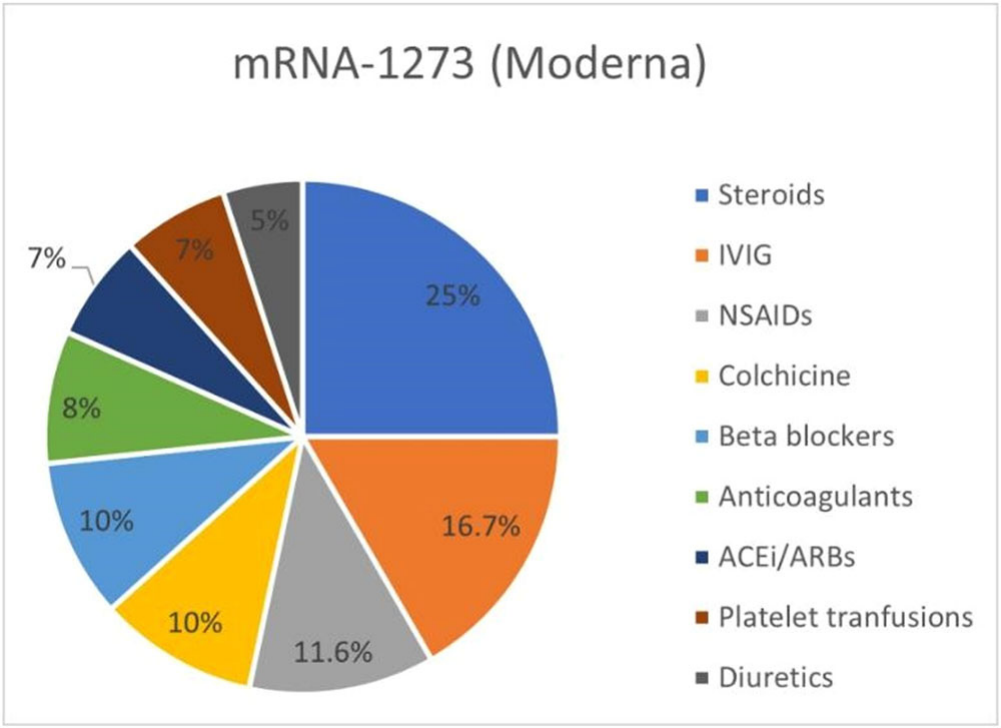
Treatment options of cardiovascular complications in COVID‐19 mRNA‐1273 (Moderna) vaccine cohort. COVID‐19, coronavirus disease 2019.

**Figure 5:**
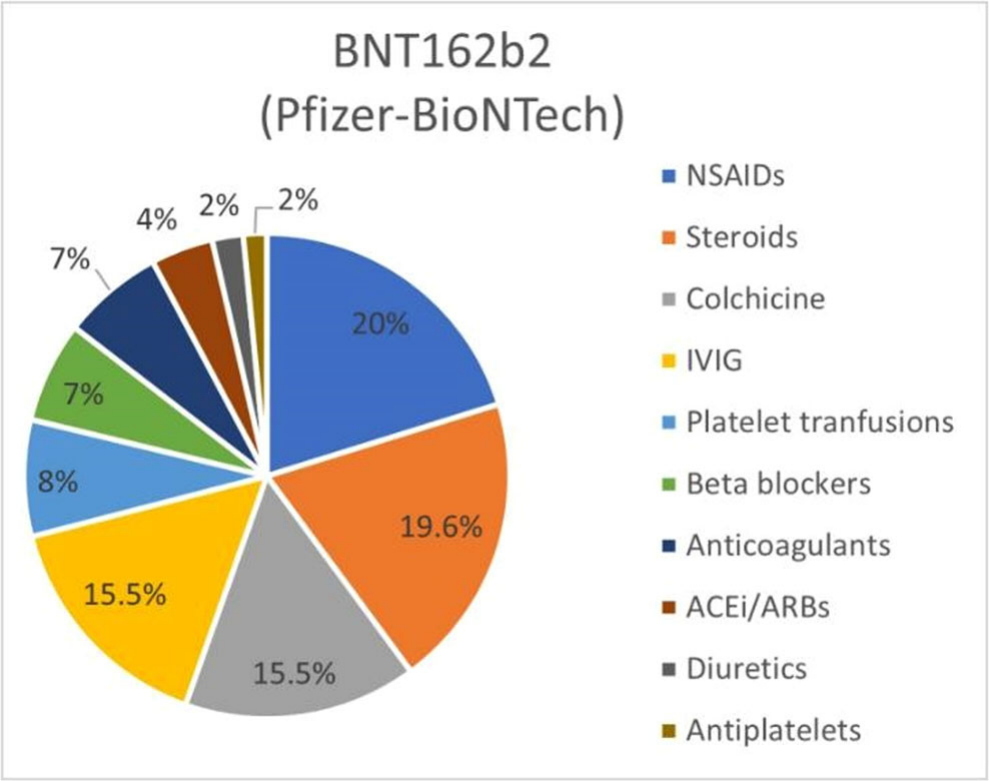
Treatment options of cardiovascular complications in COVID‐19 mRNA‐BNT162b2 (Pfizer−BioNTech) vaccine cohort. COVID‐19, coronavirus disease 2019.

Treatment options for postvaccination critical cases comprised steroids including corticosteroids, prednisone, methyl prednisone, and dexamethasone (25% for mRNA‐1273; 19.6% for BNT162b2), NSAIDs such as Ibuprofen, aspirin, acetylsalicylic acid, and ketorolac (11.6% for mRNA‐1273; 20% for BNT162b2), colchicine (10% for mRNA‐1273; 15.5% for BNT162b2), IVIGs (16.7% for mRNA‐1273; 15.5% for BNT162b2) as shown in Figures 4 and 5.

## 4 DISCUSSION

This systematic review evaluated the rare CV complications that have occurred due to mRNA vaccines and revealed that the highest complication reported combined for both mRNA vaccines was thrombosis. Thrombocytopenia has also been widely recorded and was the second‐highest adverse event to occur. Our analysis also demonstrated a moderate frequency of vascular adverse events among which, stroke was most reported and was overall, the third‐highest event to be documented. Myocarditis was the next commonly occurring complication and had the highest cases reported among all cardiac events. Other cardiac events included MI, arrhythmia, pericarditis, and cardiogenic shock. Cardiogenic shock was the lowest reported adverse effect among all events.

The mRNA vaccines have been graded as the most effective (approximately 94%) due to their strong immunogenicity and effective presentation of SARS‐CoV‐2 antigens to the immune system. Real‐world incidence of adverse reactions after mRNA vaccines has also remained lower than that concluded from clinical trials. Several systematic reviews have been published since the onset of the SARS‐CoV‐2 infection evaluating the relation between CV conditions and COVID‐19 outcomes. Cardiac complications have been observed to be the most frequently occurring complication while CV disease is the most common comorbidity.^15^ A few studies have explored the incidence of thrombosis, thrombocytopenia, and various vascular events. However, the evolving situation with the emergence of new SARS‐CoV‐2 variants requires that the focus of healthcare experts be on investigating the course of any adverse event reported concerning the vaccination phase. We presume that a thorough analysis of all adverse events arising with mRNA vaccination is necessary to know the details of these events and adopt measures to mitigate the difficulties accordingly. Recent studies analyzing complications occurring post‐mRNA vaccination have focused on a single or few complications while our study has extensively reviewed literature published so far and has considered all potential studies. For the short‐term knowledge regarding adverse effects of mRNA vaccination,^16^ our findings provide a comprehensive insight into the unfavorable sequel that has been reported and stratify them based on the different types of mRNA vaccines. This can be possibly helpful in better understanding and predicting the intricacies of mRNA vaccine inoculation.

Thrombotic events were the most common complication from our data for both vaccines overall and affected individuals displayed symptoms such as severe headache, dizziness, visual disturbances, fever, and shortness of breath.^17^ Previous studies have identified cerebral venous sinus thrombosis, splanchnic venous system thrombosis, pulmonary embolism, DVT, or acute arterial thrombosis as sites of thrombosis.^17^ As recent literature shows, these lipid nanoparticles‐mRNA‐based vaccines (Pfizer/BioNTech and Moderna) have caused rare cases of DVT, and generally, healthy individuals have presented with acute atypical thrombi. Combined descriptive analysis for the vaccines BNT162b2, mRNA‐1273, and ChAdOx1 nCov‐19 (AstraZeneca) was reported to the WHO Global Database for Individual Case Safety Reports (VigiBase) from December 13, 2020 to March 16, 2021, stated a rate of 0.21 cases of thrombotic events including CVT per 1 million individual vaccinated days.^18^ Our results are comparable to an analysis of safety surveillance data involving various US health plans for the mRNA vaccine.^19^

Thrombocytopenia, the second most reported complication in our review, has also been reported in recent studies. Atypical manifestations of thrombocytopenia presented alongside thrombosis, collectively termed VITT, have been found. Following mRNA immunization, incidence rates of VITT have been reported to be within anticipated, background levels. Our findings, however, further this knowledge by revealing a great frequency of thrombosis and thrombocytopenia occurring, higher than any other complication.^17^ The time of presentation of thrombus and concurrent thrombocytopenia is 6−24 days after the administration of the first dose mRNA (Pfizer/BioNTech and Moderna) vaccines, which corresponds with the time length presumed for the onset of the first symptom of the respective complications for the two vaccines in our paper. Elrashdy et al. propose that the mechanism underlying VITT might be the interaction between RNA molecules and platelets/platelet factor 4 (PF4). Other host conditions such as the history of thrombosis, susceptibility to thrombi, smoking, and taking medications that can cause autoimmunity have previously resulted in VITT.^20^ Recent studies assessing thrombocytopenia arising with mRNA vaccines found the cases to be reported in less than 2 weeks after vaccination.^18^ This period between vaccination and complication to surface overlaps with the time of symptom onset divulged in our results for all complications; 4.8 and 5.6 days for Moderna and Pfizer−BioNTech, respectively. Moreover, preliminary data on the relation between thrombocytopenia and mRNA vaccines had not signified any association.^18^ Given the outcome of the high incidence of thrombocytopenia in our results, it warrants further investigation to detect any correlation.

A high number of outcome events during the 21‐day risk interval have been inferred for ischemic stroke—the next most frequent complication.^19^ Additionally, an observational study that prospectively analyzed individuals who had recently received mRNA‐1273 (Moderna) or BNT162b2 (Pfizer−BioNTech) (*n* = 1,398,074) reported 246 cases of acute ischemic stroke among patients who developed cerebrovascular disorders (*n* = 286).^21^ Myocarditis, which is the fourth most common event in our study, has been increasingly reported in studies evaluating the safety of mRNA‐1273 (Moderna) and BNT162b2 (Pfizer−BioNTech), and most cases occur after the second dose of these vaccines.^22^ COVID‐19 vaccine‐associated myocarditis usually is transient and self‐limiting.^23^ Postvaccination cases of myocarditis/pericarditis are reported by Klein et al.^19^ of which 82% required hospitalization at a median length of 1 day. A meta‐analysis evaluating real‐world data from the VAERS, managed by the CDC and FDA of the United States of America, found 2−3 cases per million doses of myocarditis/pericarditis as adverse drug reaction in RNA‐based vaccines.^24^ The European Medicines Agency has officially reported myocarditis as a side effect post‐RNA vaccination, which is predominantly found among males, corresponding to 1.60 cases/million doses for Pfizer−BioNTech and 3.04 cases/million doses for Moderna in the region.^25^

Certain events, notably thromboembolism and myocarditis/pericarditis, may result in death.^24^ In our analysis, the overall mortality for each vaccine type is relatively very low in comparison to the number of events reported for the corresponding mRNA vaccine. For management of patients with damaging effects postvaccination, NSAIDs (Ibuprofen, aspirin, acetylsalicylic acid, ketorolac) were most used, followed by steroids (corticosteroids, prednisone, methyl prednisone, dexamethasone), colchicine, and IVIG. Das et al. also report NSAIDs alone to be primarily used, and NSAIDS with colchicine and NSAIDs with steroids to be the next resort for treating CV events such as myocarditis.^23^ Analogous to our analysis, ST‐segment elevation was the most common abnormal parameter, as stated by a study evaluating myocarditis post‐RNA vaccination.^23^ Diffuse ST changes and echocardiographic dysfunction are observed in our results and studies elsewhere.^23^

When differentiating adverse events for each mRNA vaccine, stroke was the highest reported complication after any dose for individuals receiving mRNA‐1273 (Moderna) vaccination. MI, pulmonary embolism, and myocarditis were the next commonly reported events. While COVID‐19 itself is a risk factor for stroke, current literature delineates the phenomenon of CVT and ischemic stroke associated with VITT occurring.^26^ Young patients with ischemic stroke have mostly presented, post‐ChAdOx1 nCoV‐19 vaccine,^27^ while our findings, that reported stroke after mRNA‐1273 vaccination, are new considering current evidence. There is a paucity of literature that details stroke‐related events following mRNA‐1273 inoculation, and while our results might indicate an underlying link, Koh et al.^21^ report acute ischemic stroke post‐mRNA vaccination to be coincidental. Moreover, the authors when discerning a plausible link between RNA‐based vaccines including mRNA‐1273, and acute ischemic stroke by assessing patients least likely to develop the condition found no pattern related to stroke etiology.^21^ For BNT162b2 (Pfizer−BioNTech) vaccine, the highest number of CV complications was thrombosis followed by thrombocytopenia, stroke, myocarditis, and pericarditis. The results are contrary to current statistics; incidences of VITT have occurred between 1 in 125,000 and 1 in 1 million, mainly linked to the ChAdOx1‐nCoV‐19 vaccine, while only 1 reported case of DVT linked with the BNT162b2 mRNA COVID‐19 vaccine.^28^ Unlike the DNA‐borne adenoviral vaccines, low rates of VITT could be due to the changes in mRNA vaccines that diminish dampen pathogen‐associated molecular pattern sensing mechanisms lowering the immunogenic risk.^17^ However, there might be a need to revisit this proposed mechanism as other factors might be at play, resulting in a high incidence of thrombosis and thrombocytopenia. Our findings are comparable to phase 3 clinical trial results for the BNT162b2 (Pfizer–BioNTech) vaccine that has indicated a slight imbalance between the vaccinated and placebo groups with several events, including acute MI and cerebrovascular accidents (stroke).^29^ Stroke was also a recurring event in our analysis, however, a study based on the Scottish National Registry having recorded 0.82 million people who received BNT162b2 concluded no relationship exists between this vaccine type and stroke.^21^ Conversely, a study retrieving statistics from the English National Immunization (NIMS) Database of COVID‐19 constituting a cohort of more than 10 million people having received first dose of BNT162b2 deduced a positive association with BNT162b2 vaccine and hemorrhagic stroke.^30^ Myocarditis was a common complication after the aforementioned CV events and Israel, which has led the vaccination race worldwide, has reported cases of myocarditis post‐BNT162b2 (Pfizer−BioNTech) vaccination; this represents 0.001% of its population that received the second dose and was markedly high in the 16−35 years age group.^25^

The effect of COVID‐19 mRNA vaccines is eminent from our results; however, identifying the number of dosages eliciting each stratified complication and time for symptom resolution for the respective occurrence is imperative for reviewing vaccine delivery plans. Due to the studies' variable reporting, this could not be distinguished in this review. Furthermore, more studies focusing on lab parameters and biomarkers are required to employ strategies to mitigate complications and observe disease courses. Only a small proportion of our selected publications mentioned these lab findings, which, considering the number of events reported, are insufficient to establish a criterion for detecting abnormalities. Prior research has revealed myocarditis to be more common in the younger population^22^; it elucidates that dividing each condition based on age group can help discern predisposing factors and correlate vaccination and CV health. This key finding would allow strategizing vaccination in vulnerable population groups. A noteworthy limitation is the number of excluded studies as data was inconsistently presented in those and could not be categorized under our desired outcomes. Adding to this is the low to moderate quality of included studies since most are case series/case reports. However, data from electronic medical records offsets this potential constraint with a larger population size to conclude.

## 5 CONCLUSION

This systematic review provides essential data for immunization providers when evaluating the risk versus benefits of COVID‐19 mRNA vaccines on the CV system. CV events such as thrombosis, thrombocytopenia, stroke, and myocarditis frequently occur with the mRNA vaccines studied. A significant number of studies included in our review reported BNT162b2 events, which presses the need to conduct more research into the CV implications of mRNA‐1273 (Moderna) vaccine. Vaccines have a potentially life‐saving advantage, and this review merits more comprehensive studies to investigate factors that increase the susceptibility to develop deleterious CV events after mRNA vaccination. Vaccine recommendations can be reviewed considering our analysis, highlighting the need for robust post‐marketing surveillance, especially for such events that can generate findings pivotal for future evaluations that establish the safety profile of the mRNA‐1273 (Moderna) and BNT162b2 (Pfizer−BioNTech). Mortality and adverse CV events require the attention of researchers to prevent complications in immunocompromised individuals. However, the total doses administered are insufficient to draw a definitive conclusion. Future studies must report adverse events by vaccine types and changes in lab parameters of relevant outcomes pre‐ and postvaccination.

## AUTHOR CONTRIBUTIONS

**Farah Yasmin**: Conceptualization. **Hala Najeeb**: Formal analysis. **Unaiza Naeem**: Writing—original draft. **Abdul Moeed**: Writing—original draft. **Abdul Raafe Atif**: Writing—original draft. **Muhammad Sohaib Asghar**: Formal analysis. **Nayef Nimri**: Writing—review & editing. **Maryam Saleem**: Writing—review & editing. **Dhrubajyoti Bandyopadhyay**: Writing—review & editing. **Chayakrit Krittanawong**: Writing—review & editing. **Mohammed Mahmmoud Fadelallah Eljack**: Visualization. **Muhammad Junaid Tahir**: Validation. **Fahad Waqar**: Supervision.

## CONFLICT OF INTEREST STATEMENT

The authors declare no conflict of interest.

## Supporting information

Supplementary information.

## Data Availability

No new data were created or analyzed in this study. Data sharing is not applicable to this article.
