# Supplementary material for: Adverse events following COVID‐19 mRNA vaccines: A systematic review of cardiovascular complication, thrombosis, and thrombocytopenia
Source: Immun Inflamm Dis. 2023 Mar 17;11(3):e807. doi: 10.1002/iid3.807 (PMC10022421; doi:10.1002/iid3.807)
Supplement: Supplementary file 1 — Supplementary information. [file IID3-11-e807-s001.docx]

**Index**

**Supplementary table 1: All cardiac events**

**Supplementary table 2: All vascular and thrombotic events**

**Supplementary table 3: All thrombocytopenia events**

**Supplementary table 1: All cardiac events**

| **Study** | **Vaccine Type** | **Time between vaccine dose and onset of symptoms (days)** | **Presenting complication** |
| --- | --- | --- | --- |
| Ehrlich et al | BNT162b2 (Pfizer-BioNTech) | 4 | Acute lymphocytic myocarditis (n=1) |
| Khogali et al | mRNA-1273 (Moderna) | 10 | Acute perimyocarditis (n=1) |
| Abbate et al | BNT162b2 (Pfizer-BioNTech) | 1.5 | Fulminant pericarditis (n=1) |
|  |  | 4 | Myopericarditis (n=1) |
| Barda et al | BNT162b2 (Pfizer-BioNTech) | - | Myocardial infarction (n=59) Myocarditis (n=21) Pericarditis (n=27) Arrythmia (n= 254) |
| Verma et al | BNT162b2 (Pfizer-BioNTech) | 10 | Myocarditis (n=1) |
|  | mRNA-1273 (Moderna) | 14 | Myocarditis (n=1) |
| Kim et al | BNT162b2 (Pfizer-BioNTech) | 1 | Acute myocarditis (n=1) |
| Chen et al | BNT162b2 (Pfizer-BioNTech) | 4 | Cardiogenic shock (n=1) |
| King et al | mRNA-1273 (Moderna) | 5 | Myocarditis (n=3) |
|  |  | 2 |  |
|  |  | 4 |  |
|  | BNT162b2 (Pfizer-BioNTech) | 4 | Myocarditis (n=1) |
| Montgomery et al | BNT162b2 (Pfizer-BioNTech) | 2 | Myocarditis (n=7) |
|  | mRNA-1273 (Moderna) |  | Myocarditis (n=16) |
| Dionne et al | BNT162b2 (Pfizer-BioNTech) | 3.5 | Myocarditis (n=15) |
| Patrignani et al | BNT162b2 (Pfizer-BioNTech) | 4 | Acute myocarditis (n=1) |
| Ramírez-García et al | BNT162b2 (Pfizer-BioNTech) | 11 | Pericarditis (n=2) |
| Hause et al | BNT162b2 (Pfizer-BioNTech) | - | Myocarditis (n=348) |
| Hasnie et al | mRNA-1273 (Moderna) | 3 | Myopericarditis (n=1) |
| Hudson et al | BNT162b2 (Pfizer-BioNTech) | 3 | Myopericarditis (n=2) |
|  |  | 0.5 |  |
| Williams et al | mRNA-1273 (Moderna) | 1 | Perimyocarditis (n=1) |
| Starekova et al | BNT162b2 (Pfizer-BioNTech) | 2 | Acute myocarditis (n=5) |
|  | BNT162b2 (Pfizer-BioNTech) | 3 |  |
|  | BNT162b2 (Pfizer-BioNTech) | 2 |  |
|  | mRNA-1273 (Moderna) | 3 |  |
|  | mRNA-1273 (Moderna) | 3 |  |
| Vidula et al | BNT162b2 (Pfizer-BioNTech) | 4 | Acute myocarditis (n=2) |
|  | mRNA-1273 (Moderna) | 1 |  |
|  | BNT162b2 (Pfizer-BioNTech) | 4 | Stress cardiomyopathy |
|  | BNT162b2 (Pfizer-BioNTech) | 21 | Pericarditis (n=2) |
|  | BNT162b2 (Pfizer-BioNTech) | 28 |  |
| Singh et al | BNT162b2 (Pfizer-BioNTech) | 3 | Myocarditis (n=1) |
| Tajstra et al | BNT162b2 (Pfizer-BioNTech) | 0.02 | Acute ST-elevated Myocardial infarction / Acute coronary thrombosis (n=1) |
| Ishay et al | BNT162b2 (Pfizer-BioNTech) | 1 | Pericarditis (n=2) |
|  |  | 10 |  |
|  |  | 14 | Myocarditis (n=1) |
| Dickey et al | BNT162b2 (Pfizer-BioNTech) | 4 | Myocarditis (n=6) |
|  | BNT162b2 (Pfizer-BioNTech) | 3 |  |
|  | mRNA-1273 (Moderna) | 4 |  |
|  | BNT162b2 (Pfizer-BioNTech) | 2 |  |
|  | BNT162b2 (Pfizer-BioNTech) | 4 |  |
|  | BNT162b2 (Pfizer-BioNTech) | 3 |  |
| Watkins et al | BNT162b2 (Pfizer-BioNTech) | 2 | Myocarditis (n=1) |
| Kim et al | mRNA-1273 (Moderna) | 1 | Acute myocarditis (n=4) |
|  | BNT162b2 (Pfizer-BioNTech) | 1 |  |
|  | mRNA-1273 (Moderna) | 1 |  |
|  | BNT162b2 (Pfizer-BioNTech) | 1 |  |
| Park et al | BNT162b2 (Pfizer-BioNTech) | 3 | Myocarditis (n=2) |
|  |  | 2 |  |
| Mansour et al | mRNA-1273 (Moderna) | 1 | Acute myocarditis (n=2) |
| Minocha et al | BNT162b2 (Pfizer-BioNTech) | 1 | Acute myocarditis (n=1) |
| Rosner et al | BNT162b2 (Pfizer-BioNTech) | 3 | Myocarditis (n=6) |
|  | mRNA-1273 (Moderna) | 4 |  |
|  | BNT162b2 (Pfizer-BioNTech) | 7 |  |
|  | BNT162b2 (Pfizer-BioNTech) | 2 |  |
|  | BNT162b2 (Pfizer-BioNTech) | 3 |  |
|  | BNT162b2 (Pfizer-BioNTech) | 3 |  |
| Larson et al | mRNA-1273 (Moderna) | 3 | Myocarditis (n=8) |
|  | mRNA-1273 (Moderna) | 3 |  |
|  | BNT162b2 (Pfizer-BioNTech) | 2 |  |
|  | BNT162b2 (Pfizer-BioNTech) | 3 |  |
|  | BNT162b2 (Pfizer-BioNTech) | 3 |  |
|  | BNT162b2 (Pfizer-BioNTech) | 2 |  |
|  | BNT162b2 (Pfizer-BioNTech) | 4 |  |
|  | mRNA-1273 (Moderna) | 2 |  |
| Bardenheier et al | BNT162b2 (Pfizer-BioNTech) | 2 | Acute myocardial infarction (n=1) |
| Abou Mouch | BNT162b2 (Pfizer-BioNTech) | 3 | Myocarditis (n=6) |
|  |  | 1 |  |
|  |  | 2 |  |
|  |  | 16 |  |
|  |  | 1 |  |
|  |  | 3 |  |
| Welsh et al | BNT162b2 (Pfizer-BioNTech) | - | Myocardial infarction (n=1) |
| Walad et al | BNT162b2 (Pfizer-BioNTech) | 4 | Pericarditis (n=3) |
|  |  | 2 |  |
| Lee et al | BNT162b2 (Pfizer-BioNTech) | - | Myocarditis (n=1) |
| Deb et al | mRNA-1273 (Moderna) | 0.25 | Myocarditis (n=1) |
| Muthukumar et al | mRNA-1273 (Moderna) | - | Myocarditis (n=1) |
| Diaz et al | BNT162b2 (Pfizer-BioNTech) | - | Myocarditis (n=9) Pericarditis (n=23) |
|  | mRNA-1273 (Moderna) |  | Myocarditis (n=11) Pericarditis (n=12) |
| Levin et al | BNT162b2 (Pfizer-BioNTech) | 1 | Myocarditis (n=7) |
|  |  | 1 |  |
|  |  | 1 |  |
|  |  | 5 |  |
|  |  | 2 |  |
|  |  | 5 |  |
|  |  | 2 |  |
| El-Sawalhy et al | BNT162b2 (Pfizer-BioNTech) | 12 | Non ST-elevated myocardial infarction (n=1) |
| Tailor et al | mRNA-1273 (Moderna) | 4 | Acute myocarditis (n=1) |
| McLean et al | BNT162b2 (Pfizer-BioNTech) | 2.5 | Myopericarditis (n=1) |
| D'Angelo et al | BNT162b2 (Pfizer-BioNTech) | 3.1 | Myopericarditis (n=1) |
| Chamling B et al | BNT162b2 (Pfizer-BioNTech) | 10 | ST-elevated Myocardial infarction/Auto-immune myocarditis (n=2) |
|  |  | 3 |  |
| Habib et al | BNT162b2 (Pfizer-BioNTech) | 3 | Acute myocarditis (n=1) |
| Smadja et al | BNT162b2 (Pfizer-BioNTech) | - | Acute myocardial infarction (n=238) Acute myocardial infarction and pulmonary embolism (n=4) Thrombocytopenia associated with myocardial infarction (n=3) |
|  | mRNA-1273 (Moderna) | - | Acute myocardial infarction (n=67) |
| Marshall et al | BNT162b2 (Pfizer-BioNTech) | 2 | Acute Myocarditis (n=4) |
|  |  | 3 |  |
|  |  | 3 |  |
|  |  | 3 |  |
|  |  | 3 | Myopericarditis (n=3) |
|  |  | 2 |  |
|  |  | 3 |  |
| Ammirati et al | BNT162b2 (Pfizer-BioNTech) | 3 | Acute myocarditis (n=1) |
| Bautista et al | BNT162b2 (Pfizer-BioNTech) | - | Acute myocarditis (n=1) |
| Albert et al | mRNA-1273 (Moderna) | 4 | Myocarditis (n=1) |
| Snapiri et al | BNT162b2 (Pfizer-BioNTech) | 3 | Perimyocarditis (n=7) |
|  |  | 1 |  |
|  |  | 2 |  |
|  |  | 3 |  |
|  |  | 1 |  |
|  |  | 2 |  |
|  |  | 3 |  |

**Supplementary table 2: All vascular and thrombotic events**

| **Study** | **Vaccine Type** | **Time between vaccine dose and onset of symptoms (days)** | **Presenting complication** |
| --- | --- | --- | --- |
| Wiest et al | mRNA-1273 (Moderna) | 10 | Pulmonary embolism (n=1) |
| Hippisley-Cox et al | BNT162b2 (Pfizer-BioNTech) | - | Venous thromboembolism (n=2054) Arterial thrombosis (n=9473) |
| Barda et al | BNT162b2 (Pfizer-BioNTech) | - | Deep vein thrombosis (n=39) Other thrombosis (n=12) Pulmonary embolism (n=10) Intracranial hemorrhage (n=13) |
| Andraska et al | mRNA-1273 (Moderna) | 2 | Pulmonary embolism (n=1) |
|  |  | 3 | Pulmonary embolism (n=1) Deep vein thrombosis (n=1) |
|  |  | 3 | Deep vein thrombosis (n=1) |
| Krzywicka et al | BNT162b2 (Pfizer-BioNTech) | 7 | Cerebral venous sinus thrombosis (n=25) |
|  | mRNA-1273 (Moderna) |  | Cerebral venous sinus thrombosis (n=1) |
| Schulz et al | BNT162b2 (Pfizer-BioNTech) | - | Cerebral venous sinus thrombosis (n=8) Ischemic stroke (n=1) |
| Carli et al | BNT162b2 (Pfizer-BioNTech) | 1 | Deep vein thrombosis (n=1) |
| Bae et al | BNT162b2 (Pfizer-BioNTech) | - | Hypertension (n=10) Hypotension (n=10) |
| Fan et al | BNT162b2 (Pfizer-BioNTech) | 1 | Cerebral venous sinus thrombosis (n=3) |
|  |  | 9 |  |
|  |  | 8 |  |
| Zakaria et al | BNT162b2 (Pfizer-BioNTech) | 16 | Cerebral venous sinus thrombosis (n=1) |
| Dias et al | BNT162b2 (Pfizer-BioNTech) | 6 | Cerebral venous thrombosis (n=2) |
|  |  | 3 |  |
| Al-Maqbali et al | BNT162b2 (Pfizer-BioNTech) | 7 | Deep vein thrombosis/Pulmonary embolism (n=1) |
| Bardenheier et al | BNT162b2 (Pfizer-BioNTech) | 8 | Venous thromboembolism (n=2) |
|  |  | 12 |  |
| Athyros et al | mRNA-1273 (Moderna) | 3 | Intracranial hemorrhage (n=1) |
| Welsh et al | BNT162b2 (Pfizer-BioNTech) | - | Pulmonary embolism (n=1) |
| Meylan et al | BNT162b2 (Pfizer-BioNTech) | - | Hypertension (n=8) |
|  | mRNA-1273 (Moderna) |  | Hypertension (n=1) |
| Lee et al | BNT162b2 (Pfizer-BioNTech) | - | Pulmonary embolism (n=1) |
| Pawloski et al | BNT162b2 (Pfizer-BioNTech) |  | Cerebral venous sinus thrombosis (n=3) |
| Cari et al | BNT162b2 (Pfizer-BioNTech) | - | Cerebral venous sinus thrombosis (n=58) |
| Gerber et al | BNT162b2 (Pfizer-BioNTech) | 5 | Microvascular small bowel thrombosis (n=1) |
| El-Sawalhy et al | BNT162b2 (Pfizer-BioNTech) | 5 | Pulmonary embolism (n=2) |
|  |  | 12 |  |
|  |  | 16 | Pulmonary embolism (n=1) Deep vein thrombosis (n=1) |
| Smadja et al | BNT162b2 (Pfizer-BioNTech) | - | Pulmonary embolism (n=211) Lower limb thrombosis (n=111) Cerebral venus sinus thrombosis (n=3) Cerebral venous thrombosis (n=1) Undetermined venous thrombotic event (n=42) Stroke and MI (n=2) Others VTE (n=13) Stroke (n=561) Other ATE (n=12) Acute myocardial infarction and pulmonary embolism (n=4) Stroke and pulmonary embolism (n=3) Stroke and lower limb ischemia (n=1) Arterio-venous fistula thrombosis (n=1) Arterial limb ischemia and lower limb thrombosis (n=1) Thrombocytopenia associated with pulmonary embolism (n=2) Thrombocytopenia associated with myocardial infarction (n=3) Thrombocytopenia associated with stroke (n=13) Thrombocytopenia linked to thrombotic thrombocytopenic purpura (n=7) |
|  | mRNA-1273 (Moderna) |  | Pulmonary embolism (n=61) Lower limb thrombosis (n=13) Cerebral venus sinus thrombosis (n=3) Undetermined venous thrombotic event (n=10) Others VTE (n=1) Stroke (n=173) Other ATE (n=10) Stroke and MI (n=3) Stroke and lower limb ischemia (n=1) Arterial and venous thrombosis (n=1) |
| Sangli et al | mRNA-1273 (Moderna) | 10 | Deep vein thrombosis (n=1) Pulmonary embolism (n=1) Cerebral venous sinus thrombosis (n=1) |
| Simpson et al | BNT162b2 (Pfizer-BioNTech) | - | Arterial thrombotic events (n=1603) Venous thrombotic events (n=421) |
| Tajstra et al | BNT162b2 (Pfizer-BioNTech) | 0.02 | Acute coronary thrombosis (n=1) |

**Supplementary table 3: All thrombocytopenia events**

| **Study** | **Vaccine Type** | **Time between vaccine dose and onset of symptoms (days)** | **Presenting complication** |
| --- | --- | --- | --- |
| Hippisley-Cox et al | BNT162b2 (Pfizer-BioNTech) | - | Thrombocytopenia (n=1010) |
| Barda et al | BNT162b2 (Pfizer-BioNTech) | - | Thrombocytopenia (n=56) |
| Akiyama et al | BNT162b2 (Pfizer-BioNTech) | 12 | Immune thrombocytopenic purpura (n=1) |
| Hines et al | mRNA-1273 (Moderna) | 14 | Immune thrombocytopenic purpura (n=1) |
| King et al | BNT162b2 (Pfizer-BioNTech) | 0.5 | Immune thrombocytopenic purpura (n=1) |
| Waqar et al | BNT162b2 (Pfizer-BioNTech) | 7 | Thrombotic thrombocytopenic purpura (n=1) |
| Ganzel et al | BNT162b2 (Pfizer-BioNTech) | 14 | Immune thrombocytopenic purpura (n=1) |
| de Brujin et al | BNT162b2 (Pfizer-BioNTech) | 14 | Thrombotic thrombocytopenic purpura (n=1) |
| Fueyo-Rodriguez et al | BNT162b2 (Pfizer-BioNTech) | 0.5 | Immune thrombocytopenic purpura (n=1) |
| Welsh et al | BNT162b2 (Pfizer-BioNTech) | 7 | Thrombocytopenia (n=14) |
|  | mRNA-1273 (Moderna) |  | Thrombocytopenia (n=13) |
| Malayala et al | mRNA-1273 (Moderna) | 2 | Thrombocytopenia (n=1) |
| Lee et al | BNT162b2 (Pfizer-BioNTech) | 5 | Thrombocytopenia (n=13) |
|  | mRNA-1273 (Moderna) |  | Thrombocytopenia (n=12) |
| Toom et al | mRNA-1273 (Moderna) | 14 | Familial thrombocytopenia flare-up (n=1) |
| Cari et al | BNT162b2 (Pfizer-BioNTech) | - | Thrombocytopenia (n=219) |
| Tarawaneh et al | BNT162b2 (Pfizer-BioNTech) | 3 | Immune thrombocytopenic purpura (n=1) |
| Jasaraj et al | BNT162b2 (Pfizer-BioNTech) | 8 | Immune thrombocytopenic purpura (n=1) |
| Jawed et al | BNT162b2 (Pfizer-BioNTech) | 18 | Immune thrombocytopenic purpura flare-up (n=1) |
| Radwi et al | BNT162b2 (Pfizer-BioNTech) | 9 | Acquired hemophilia A (n=1) |
| Smadja et al | BNT162b2 (Pfizer-BioNTech) | - | Thrombocytopenia associated with pulmonary embolism (n=2) Thrombocytopenia associated with myocardial infarction (n=3) Thrombocytopenia associated with stroke (n=13) Thrombocytopenia linked to thrombotic thrombocytopenic purpura (n=7) |
